# P77 Changes in total antimicrobial consumption and pattern of UK AWaRe category utilization following multidisciplinary antimicrobial guideline review at Royal Cornwall Hospitals NHS Trust

**DOI:** 10.1093/jacamr/dlaf230.084

**Published:** 2025-12-04

**Authors:** Daniel Hearsey, Kathleen Bamford, Andree Evans, Aikaterini Ainarozidou, Chanaka Silva, Azhar Iqbal, Melissa Baxter, Gemma Vanstone, Ollie Lloyd, Nichola Leigh, Neil Powell

**Affiliations:** Pharmacy Department, RCHT, Cornwall, UK; Microbiology Department, RCHT, Cornwall, UK; Microbiology Department, RCHT, Cornwall, UK; Microbiology Department, RCHT, Cornwall, UK; Microbiology Department, RCHT, Cornwall, UK; Microbiology Department, RCHT, Cornwall, UK; Microbiology Department, RCHT, Cornwall, UK; Microbiology Department, RCHT, Cornwall, UK; Infectious Disease Department, RCHT, Cornwall, UK; Pharmacy Department, RCHT, Cornwall, UK; Pharmacy Department, RCHT, Cornwall, UK

## Abstract

**Background:**

The Royal Cornwall Hospitals NHS Trust (RCHT) is a large acute trust with approximately 750 beds and an active antimicrobial stewardship programme. The UK National Action Plan aims to reduce total antimicrobial consumption with 70% of all antibiotic prescribing from the ‘Access’ category of the UK AWaRe classification.^1^

**Objectives:**

To assess the impact the changes to the RCHT’s empirical antimicrobial guidelines had on total antimicrobial consumption and utilization of antibiotics in both the Access and non-Access (Watch and Reserve) groups.^2^

**Methods:**

RCHT’s empirical antimicrobial guidelines were updated in February 2024 on Eolas (previously Microguide) following review by the RCHT Infection Specialists team (Infectious Diseases consultant, Consultant Microbiologists and Antimicrobial Stewardship Pharmacists) and engagement from the clinical specialist teams within the trust. The guideline review removed ‘Watch’ and ‘Reserve’ antibiotics from the guidelines and replaced them with ‘Access’ antibiotics, where it was felt appropriate for empirical management. In addition, doses of amoxicillin, doxycycline and flucloxacillin for empirical treatment of respiratory infections and cellulitis respectively, were increased in line with national recommendations.^3^ Antimicrobial consumption data were extracted from RxInfo Define platform.^4^ Baseline consumption data for 2022–23 and 2023–24 (prior to the guideline changes in Q4 2023–24_were compared to 12 months consumption data post-guideline implementation (Q1–Q4 2024–25) to determine the impact of the guideline changes on antibiotic consumption.

**Results:**

Total antimicrobial consumption for 2022–23 and 2023–24 were 3504 and 3674 DDDs/1000 admissions, respectively. Total consumption in 2024–25 was 3936 DDDs/1000 admissions; a 5% increase from 2022–23 to 2023/24, and a 7% increase from 2023–24 to 2024–25. Access consumption increased from 2281 DDDs/1000 admissions in 2023–24 to 2728 DDDs/1000 admissions in 2024–25, an 8% increase. Non-access (‘Watch’ and ‘Reserve’) consumption decreased from 1319 DDDs/1000 admissions in 2023–24 to 1123 DDDs/1000 admissions in 2024–25, a 7% decrease. The proportion of ‘Access’ to ‘non-Access’ antibiotics prescribed in 2022–23 and 2023–24 were 60% and 63%, respectively, increasing in 2024–25 to 71%.

**Conclusions:**

A multidisciplinary approach to incorporating the UK AWaRe guidance has increased the proportion of ‘Access’ antibiotics and reduced the proportion of ‘Watch’ and ‘Reserve’ antibiotics.^5^ Access antibiotic consumption increased likely due to the replacement of single ‘Watch’ and ‘Reserve’ antibiotics with multiple antibiotics from the ‘Access’ category and dose increases of some Access antibiotic used for common antibiotics. There were reductions in non-Access antibiotic consumption.
